# The role of long non-coding ribonucleic acid HOXA11-AS in endometriosis therapy

**DOI:** 10.1186/s12958-025-01420-0

**Published:** 2025-06-02

**Authors:** Ramanaiah Mamillapalli, Nimisha Gawde, Madeline Fay, Rula Atwani, Irene Moridi, Hugh S. Taylor

**Affiliations:** Department of Obstetrics, Gynecology and Reproductive Sciences, Yale School of Medicine, New Haven, CT 06520 USA

**Keywords:** HOXA11-AS, HOXA11, Endometriosis, Women’s health, Progestin, Endometrium

## Abstract

**Objective:**

To investigate the function of HOXA11-antisense long non-coding RNA (HOXA11-AS) in endometriosis treatment response.

**Methods:**

Tissue samples (ectopic and eutopic endometrium) were obtained from surgically diagnosed subjects with endometriosis (*n* = 15) and controls (*n* = 11) without endometriosis after treatment with a progestin. RNA was extracted from these tissues; cDNA was prepared and lncRNA HOXA11-AS levels were measured by quantitative real-time polymerase chain reaction (RT-qPCR). Immortalized endometrial stromal cells from an endometriosis patient (ENDO cell line) were cultured and transfected by HOXA11-AS plasmid and potential target genes were analyzed by RT-qPCR.

**Results:**

Progestin therapy led to lower lncRNA *HOXA11-AS* expression. HOXA11-AS was most decreased in ectopic endometriotic lesions, lower by 81% compared to eutopic endometrium from women with endometriosis. There was no difference in progestin response between eutopic endometrium in endometriosis and normal endometrium from controls. A HOXA11-AS plasmid was used to increase HOXA11-AS expression in an endometriotic cell line. Increased HOXA11-AS led to a significant increase in the expression of genes ITGB3, AKT1, MMP2, and MMP9, which have a role in cell proliferation and tumorigenesis. HOXA11-AS also upregulated the mRNA levels of tumor suppressor and apoptotic regulatory genes PTEN, BCL2 and Caspase3.

**Conclusions:**

HOXA11-AS is a critical regulator of normal endometrial development. HOXA11-AS is elevated in endometriosis contributes to its pathophysiology. This long non-coding RNA was decreased in women undergoing endometriosis treatment with progestins. HOXA11-AS regulated several key drivers of disease and repression during treatment likely has a central role in preventing growth and invasion of endometriosis.

**Supplementary Information:**

The online version contains supplementary material available at 10.1186/s12958-025-01420-0.

## Introduction

HOXA11-antisense (HOXA11-AS), also known as NCRNA00076, is a long non-coding RNA (lncRNA) previously implicated in endometriosis and other reproductive disease. Long non-coding RNAs are a subset of non-coding RNAs (ncRNAs) that are abundant in human genome transcripts [1]. Out of the 28,000 lncRNAs identified in human genome, only a few have been well characterized with known biological functions [2]. LncRNAs are linear molecules longer than 200 nucleotides in length that do not code for proteins; however they are regulators of protein-coding genes, and their tissue specific expression levels are generally lower than protein-coding mRNAs [3]. LncRNAs have a role in the expression and regulation of various genes by epigenetic, transcriptional, and post-transcriptional modulation [4]. LncRNAs play a crucial role in multiple biological processes including cell proliferation and differentiation, migration, invasion, X chromosome inactivation (Xi), stem cell pluripotency, cell cycle progression, cellular reprogramming, apoptosis, and organogenesis [5–7]. Dysregulation of lncRNA expression is implicated in human diseases including cancers [8, 9]. These lncRNAs may act either as a tumor accelerator or as a tumor suppressor. Therefore, lncRNAs are emerging as key molecular regulators in the pathophysiology of many human cancers. Modulation of lncRNAs may be helpful in the prevention and treatment of cancers as well as other diseases [10].

HOXA11-AS is located on chromosome at 7p15.2 in the HOXA gene cluster, one of the four clusters of mammalian HOX genes [11, 12]. The HOXA cluster contains three lncRNAs HOXA11- AS, HOXA10-AS, and HOXA (HOTTIP). Among these three, HOXA11-AS acts as an epigenetic modulator that functions as an oncogene or tumor suppressor playing a vital role in carcinogenic cellular processes including differentiation, proliferation, migration, and invasion [13]. Aberrant expression of HOXA11-AS has been implicated in the progression and prognosis of malignancies, including small cell lung cancer, osteosarcoma, uveal melanoma, glioma, hepatocellular carcinoma, gastric, breast, colorectal cancers, and glioblastoma [14–16]. It also serves as a prognosticator for cervical cancer with higher expression correlating with poorer survival rates [17]. Similarly, HOXA11-AS promotes cell proliferation, invasion and migration of the serous ovarian cancer [18]. In the uterus, HOXA11-AS expression is reciprocal to HOXA11 expression, HOXA11-AS expression being maximal in the proliferative phase [19]. The HOXA11 antisense transcript promotes endometrial proliferation and is repressed by progesterone [20]. Overexpression attenuates endometrial decidualization, leading to recurrent embryonic implantation failure [21, 22].

Endometriosis is an estrogen dependent chronic inflammatory gynecological disorder characterized by the growth of endometrial cells outside the uterus, most commonly in the pelvis. Endometriosis affects approximately 10% of women of reproductive age and causes both chronic pelvic pain and infertility [23]. Several protein coding mRNAs, respective proteins, and their signaling pathways have been implicated in the initiation, development, and progression of endometriosis [24]. Besides conventional protein coding RNAs, non-coding RNAs, including lncRNAs such as H19, AC002454.1, LINC01279, and FAS-AS1, have been implicated in the pathophysiology of endometriosis [25–28]. It is likely that lncRNAs play a crucial role in the pathophysiology of endometriosis, but their potential functional roles are still unknown. HOXA11-AS regulates pregnancy by preventing decidualization [22]. Progesterone normally suppresses HOXA11-AS in the secretory phase allowing decidualization [22]. HOXA11-AS RNA is increased in endometriosis where it likely prevents differentiation and allows continued growth and invasion [29]. Here, we describe the HOXA11-AS expression pattern in women with endometriosis treated for endometriosis with progestins and the differential expressions of its target genes following its expression in endometriosis cells in vitro. Identifying the target molecules and the signaling pathways regulated by HOXA11-AS in the pathophysiology of endometriosis may provide diagnostic and therapeutic strategies for endometriosis disease management.

## Materials and methods

### Study population

Samples were collected from subjects who underwent laparoscopy for suspected benign gynecological conditions, including endometriosis. Approval for the collection of the specimens was obtained from the Institutional Review Board (IRB) at Yale University School of Medicine (New Haven, CT). Written consent was obtained from patients undergoing surgery for suspected benign indications between January 2021 and December 2022. Inclusion criteria were age 20–50 years, use of progestin based hormonal therapy for at least 3 months before surgery, and no evidence of other inflammatory disease or cancer. The progestin-based regiment typically consisted of a combined oral contraceptive, however those receiving systemic progestins alone were not excluded. Subjects were classified into the disease group if visual and pathology findings from surgery confirmed the presence of endometriosis (*N* = 15) or the control group if surgery found no endometriosis. Participants were assigned to the endometriosis group after pathologic confirmation of the excised tissue. Staging was determined using the revised American Society of Reproductive Medicine (rASRM) classification [30]. Subjects with histologically confirmed endometriosis had mild-to-severe peritoneal and/or ovarian endometriosis (stages I to IV). Control subjects consist of women with no evidence of endometriosis or other endometrial diseases (*N* = 11). Endometrial biopsies were performed using a Pipelle catheter (Code #8200, CooperSurgical Inc., Trumbull, CT. USA). Exclusion criteria consisted of use of other hormonal or sex hormone modifying medications, postmenopausal state, pregnancy, critical anemia, hyperplasia, polyps or malignances, autoimmune disease, infectious diseases, chronic or acute inflammatory diseases. All studies were performed according to the declaration of Helsinki and approved by the Ethics Committee of the Yale University (Approval Protocol No. 2023–07113).

### Tissue collection and RNA extraction

Fresh tissue samples (ectopic endometrial lesions and eutopic endometrium) from patients with endometriosis and eutopic endometrium from control subjects were collected. The finely minced tissue was homogenized in 1 mL of TRIzol reagent (Catalog #15596018, Invitrogen, Carlsbad, CA, USA) to extract total RNA. After incubating the extracted RNA for 5 min on ice, 0.2 ml of chloroform was added to each. The samples were vortexed for 20 s, incubated at RT for 3 min, and then centrifuged at 12,000 rpm at 4 °C for 15 min. Then, the aqueous layer was transferred to a fresh tube, then RNA was precipitated by adding 0.5 ml isopropyl alcohol, incubating at RT for 10 min, and then centrifuging at 10,000 rpm for 15 min. Then the supernatant was removed, and RNA pellets were collected. Following two washes in 70% ethanol, pellets were dried and dissolved in 30–50 µL of RNase-free water. Total RNA was purified using the RNeasy cleanup kit (Catalog #74204, Qiagen, Valencia, CA, USA) as per the manufacturer’s protocol, treated using recombinant DNase (Catalog #18047019, USB, Cleveland, OH, USA) to eliminate DNA contamination, and quantified using a NanoDrop spectrophotometer. Purified RNA was immediately used for cDNA synthesis or stored at − 80 °C until later use. For cDNA synthesis, purified RNA (1000 ng) was reverse transcribed using an iScript cDNA synthesis kit (Catalog #1708891, Bio-Rad Laboratories, Inc., Hercules, CA, USA).

### HOXA11-AS plasmid preparation

HOXA11-AS plasmid was obtained from Synbio Technologies, Monmouth Junction, NJ, USA. The gene, which is 1640 base pairs (bp) [13] in length, was inserted into the vector pcDNA3.1(+) with cloning sites BamHI GGATCC and EcoRI GAATTC. The sequences of both vector pcDNA3.1(+) and HOXA11-AS gene were given in Supplementary Tables 1 and the HOXA11-AS plasmid map showed in Supplementary Fig. 1. Transformation was carried out using DH5-⍺ competent *E. coli* cells with plasmid on agar plate and the grown colonies were selected. Plasmid was amplified initially in mini cultures (3 ml) of LB broth with 100 µg/ml ampicillin (Catalog #10855-021, Gibco, Life Technologies Corporation, Grand Island, NY, USA) and mini culture was used for maxiprep plasmid isolation (250 ml culture). The QIAprep Spin Miniprep Kit (Catalog #27204, Qiagen) and HiSpeed Plasmid Maxi Kit (Catalog #12662, Qiagen, Hilden, Germany) were used for the isolation of HOXA11-antisense and vector pcDNA3.1 plasmids. Agar gel electrophoresis was performed to identify and analyze the DNA fragments of the right size for vector (5428 bp) and vector + HOXA11-AS plasmid (7039 bp) including cloning sites.

### Cell culture and transfection

A telomerase immortalized endometriotic stromal cell line (ENDO), was cultured in growth medium containing Dulbecco’s Modified Egale Medium (DMEM, Catalog #11330-032, Gibco, Life Technologies Corporation, NY, USA) with 10% fetal bovine serum (FBS, Gibco, Catalog #A56708-01) and 1% antibiotic and antimycotic (anti-anti, Catalog #15240-062, Gibco). Growth medium (500 ml) containing 445 ml of DMEM, 50 ml of FBS and 5 ml of 100x anti-anti. The Endo stable cell line was originated from the lesions of the patient with endometriosis. For transfection, 2 × 10^5^ cells per well were seeded in 6 well plates in antibiotic free media. Cells were transfected at 60% confluence in serum and antibiotic free media with the HOXA11-AS plasmid or vector plasmid (pcDNA) as a control, following the Lipofectamine 3000 reagent protocol. All transfections were performed in duplicates under sterile conditions. 24 h post-transfection, media was changed to growth medium to include serum and antibiotics. Total RNA was isolated from cells 48 h of post-transfection using the RNeasy Plus Micro Kit (Catalog #74034, Qiagen) according to the manufacturer’s protocol. RNA was quantified by Nano Drop 2000 Spectrometer. Purified RNA (1000 ng) was immediately reverse transcribed to cDNA using iScript cDNA synthesis kit (Bio-Rad Laboratories). RNA was stored at -80^o^C while cDNA was stored at -20^o^C for later use.

### Real-Time Quantitative Polymerase Chain Reaction (RT-qPCR)

Real-time quantitative PCR (real-time qPCR) was performed using SYBR Green (Bio-Rad Laboratories) and optimized in the MyiQ single-color real-time PCR detection system (Bio-Rad). Purified RNA (1000 ng) from either tissue from subjects or HOXA11-AS transfected cells (ENDO cells) was reverse transcribed into complementary DNA (cDNA) using iScript cDNA synthesis kit (Bio-Rad Laboratories, Hercules, CA). Reactions were performed in a 15 µl volume containing 1.5 µl cDNA (1:10 dilution), 1 µl primer mix, 7.5 µl master mix (SYBR^®^ Green, Catalog #1708882, BioRad Laboratories), and 5 µl water. PCR cycling conditions were as follows: polymerase activation and the initial DNA denaturation step required a temperature of 95 °C for 3 min followed by 40 cycles of 30 s denaturation at 95 °C and 20 s of annealing and extension at 57 °C. Gene expression was normalized to glyceraldehyde 3-phosphate dehydrogenase (GAPDH) as an internal control. Relative mRNA expression was calculated using the comparative cycle threshold (Ct) method (2^−ΔΔCT^) [31]. The specificity of the amplified transcript and the absence of primer-dimers were confirmed by a melting curve analysis. Primer sequences used for gene expression analysis are listed in Table 1. Primers were obtained from the W.M. Keck Oligonucleotide Synthesis Facility (Yale University, New Haven, CT). All experiments were carried out three times, with each done in duplicate.

**Table 1:** Primer sequences used for gene expression by RT-qPCR

Gene	Forward	Reverse
*BAX*	5’-CCCGAGAGGTCTTTTTCCGAG-3’	5’-CCAGCCCATGATGGTTCTGAT-3’
*BCL2*	5’-GGTGGGGTCATGTGTGTGG-3’	5’-CGGTTCAGGTACTCAGTCATCC-3’
*MMP2*	5’-TACAGGATCATTGGCTACACACC-3’	5’-GGTCACATCGCTCCAGACT-3’
*MMP9*	5’-TGTACCGCTATGGTTACACTCG-3’	5’-GGCAGGGACAGTTGCTTCT-3’
*PTEN*	5’-TGGATTCGACTTAGACTTGACCT-3’	5’-GGTGGGTTATGGTCTTCAAAAGG-3’
*AKT1*	5’-AGCGACGTGGCTATTGTGAAG-3’	5’-GCCATCATTCTTGAGGAGGAAGT-3’
*CASP3*	5’-CATGGAAGCGAATCAATGGACT-3’	5’-CTGTACCAGACCGAGATGTCA-3’
*ITGB3*	5’-GTGACCTGAAGGAGAATCTGC-3’	5’-CCGGAGTGCAATCCTCTGG-3’
*HOXA11-AS*	5’-GAGTGTTGGCCTGTCCTCAA-3’	5’- TTGTGCCCAGTTGCCTGTAT-3’
*GAPDH*	5’-TCAAGGCTGAGAACGGGAAG-3’	5’-CGCCCCACTTGATTTTGGAG-3’

### Statistical analysis

GraphPad Prism 10.0 software (GraphPad Software, La Jolla, CA, USA) was used for statistical analyses. The quantitative data were tested for normality using the Shapiro–Wilk test. Normally distributed data were evaluated using Student’s *t*-test, and Mann–Whitney *U* test was used for non-normally distributed data. A *p-*value of < 0.05 was considered statistically significant.

## Results

Among original subjects who gave written consent, three were excluded because of an unexpected comorbidity or inflammatory disease. The final number of subjects was 26, with 15 categorized as having endometriosis and 11 as controls. Table 2 shows the summary of demographics and clinical characteristics of the subjects. There was no statistically significant difference in age and body mass index between control and endometriosis groups. Approximately 85% of the surgeries were performed for pelvic pain and 15% for infertility in women with endometriosis. The control subjects had varying benign gynecological pathologies while the endometriosis group showed different stages of disease as categorized by rASRM staging of stage I, II, III, or IV. The presence of endometriosis was not known prior to surgery in this case-control study.

**Table 2:** Patient demographics and clinical characteristics

Variables	Endometriosis (*n* = 15)	Control (*n* = 11)
Age	35.11+/-7.17	40.4 +/- 10.784
Body mass index	29.195+/-8.24	38.62 +/- 9.69
Race, n%		
White	9 (60%)	8 (72%)
Black/African American	3 (20%)	2 (18%)
Hispanic	-	-
Asian	3 (20%)	1 (9%)
Others	-	-
rASRM endometriosis stage		
I	1 (6%)	-
II	1 (6%)	-
III	2 (13%)	-
IV	11 (73%)	-
Hormonal treatment		
Combined OC	13 (87%)	8 (73%)
Progestin alone	2 (13%)	3 (27%)
Control Diagnoses		
No abnormality	-	-
Leiomyoma	-	8 (72%)
Ovarian cyst	-	3 (27%)

*rASRM*, revised American Society of Reproductive Medicine

**Fig. 1:**
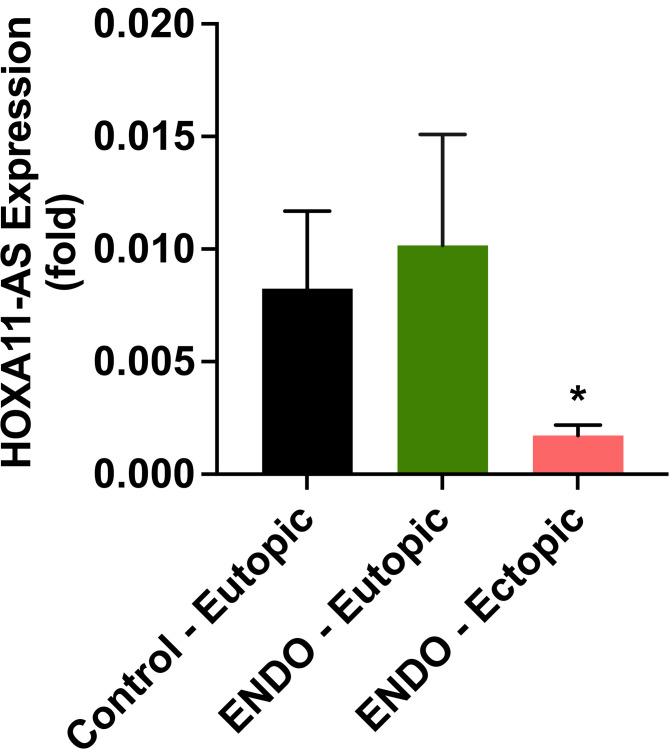
Expression of HOXA11-AS in ectopic lesions from patients with endometriosis determined by quantitative RT-qPCR. HOXA11-AS mRNA levels were downregulated in endometrium and endometriosis tissues in response to progestin treatment. There was significantly greater downregulation in the endometriotic lesions treated with progesterone compared to control eutopic and ENDO eutopic tissues. Each bar represents the means ± SEM for data from 3 individual experiments, and each experiment was performed in duplicate. *P < 0.05, statistical significance, ENDO ectopic vs. ENDO eutopic and eutopic controls

Figure 1 shows the mRNA expression levels of long non-coding RNA (lncRNA) HOXA11-AS in tissue collected from endometriosis and control subjects; both groups were treated with a progestin-based therapy consisting of a combination oral contraceptive (COC), progestin only oral contraceptive or progestin in other formulation. HOXA11-AS was significantly decreased in ectopic endometriotic lesions by 81% (*p* = 0.04) compared to eutopic endometrium from women with endometriosis and also from eutopic endometrium from control subjects as shown in Fig. 1. There was no significant difference in the expression levels of HOXA11-AS between eutopic endometrium in endometriosis and normal endometrium from controls. There were no significant changes are observed between COC (*n* = 13) and progestin only (*n* = 2) treatment groups (Supplementary Fig. 2).

**Fig. 2:**
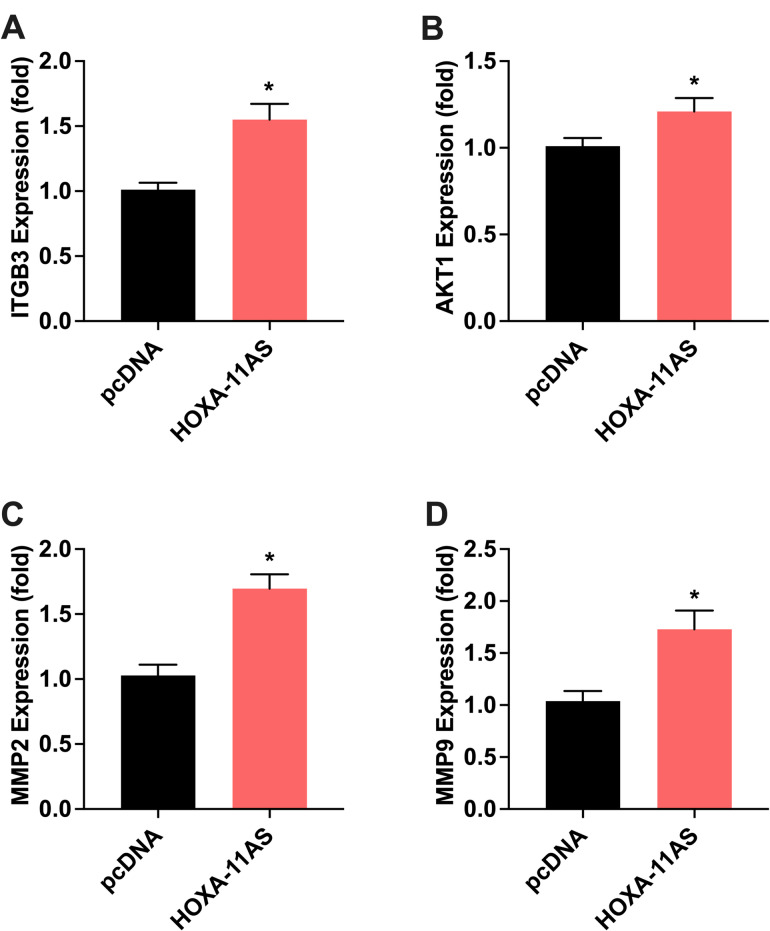
Effect of HOXA11-AS plasmid on mRNA expression levels of cell proliferative and tumorigenic genes determined by RT-qPCR in ENDO cells. (**A**) ITGB3, (**B**) AKT1, (**C**) MMP2 and (**D**) MMP9. Each bar represents the means ± SEM for data from 3 individual experiments, and each experiment was performed in duplicate. *P < 0.05, statistical significance, pcDNA (vector) vs. HOXA11-AS plasmid overexpression

**Fig. 3:**
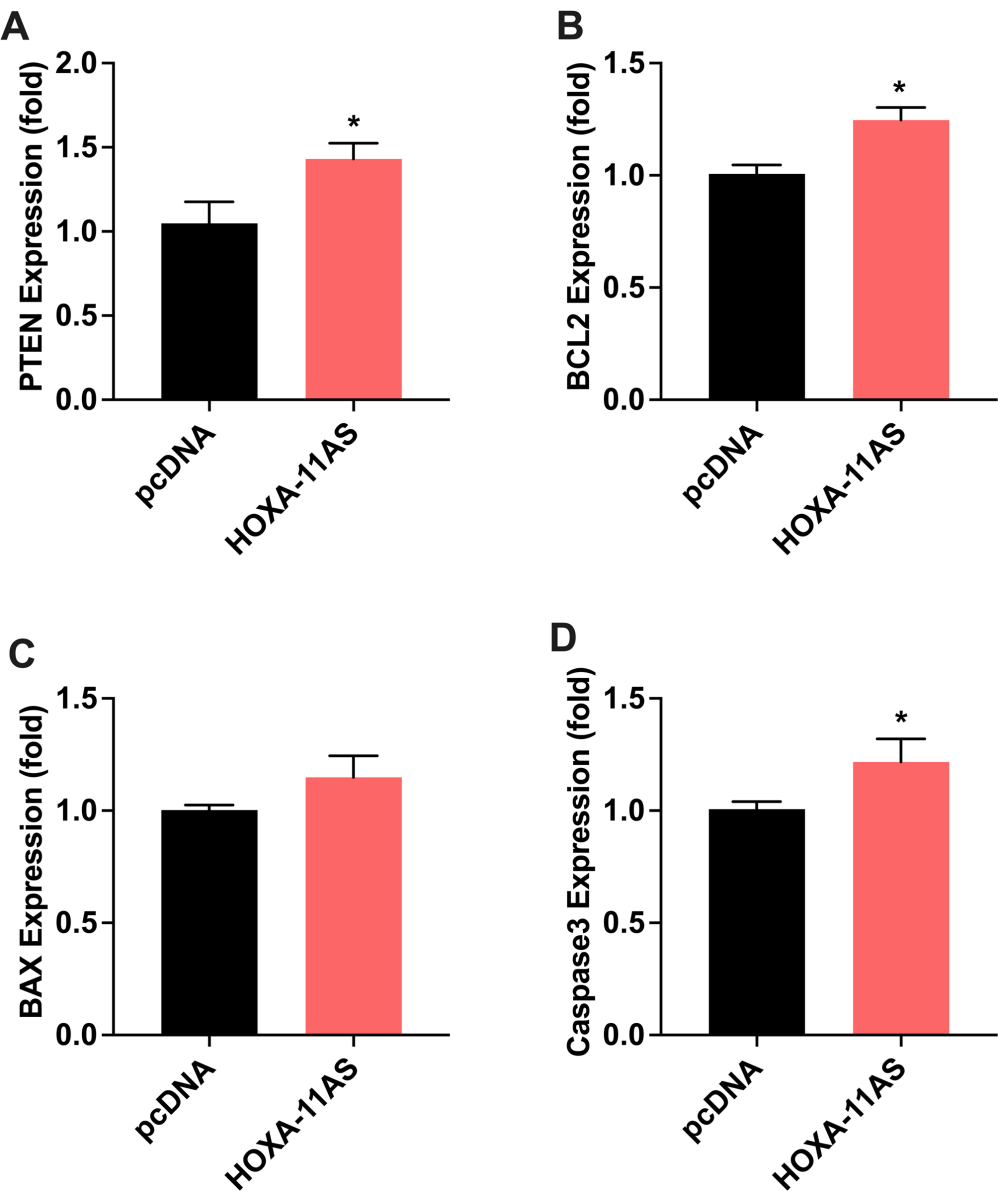
Effect of HOXA11-AS plasmid on mRNA expression levels of cell tumor suppressor and proapoptotic genes determined by RT-qPCR ENDO cells. Tumor suppressor genes PTEN (**A**), BCL2 (**B**). Proapoptotic genes BAX (**C**) and Caspase 3 (**D**). Each bar represents the means ± SEM for data from 3 individual experiments, and each experiment was performed in duplicate. *P < 0.05, statistical significance, pcDNA (vector) vs. HOXA11-AS plasmid overexpression

Next, we identified lncRNA HOXA11-AS targeted genes. To determine its ability to regulate gene expression of selected targets, we transfected HOXA11-AS plasmid in ENDO cells, a telomerase immortalized cell line developed from an endometriosis patient. We screened more than 20 genes that are predicted targets of HOXA11-AS regulation. One of the primary mechanisms of action of lncRNAs is the binding and sequestration of microRNA. We first identified all reported microRNAs that bind HOXA11-AS and then used commercially available programs to pick target genes of these microRNAs; we then selected several genes from pathways that are thought to be involved in endometriosis pathology. Genes that are involved in cell proliferation, invasion, metastasis and tumor growth were differentially expressed in HOXA11-AS transfected ENDO cells. Figure 2 shows the significant increase in mRNA levels of *ITGB3* (Fig 2 A, 1.6-fold, *p* = 0.0007), *AKT1* (Fig. 2B, 1.2-fold, *p* = 0.04), *MMP2* (Fig. 2 C, 1.6-fold, *p* = 0.0001), and *MMP9* (Fig. 2 D, 1.8- fold, *p* = 0.003). *ITGB3 plays a* role in tumor growth by reprogramming tumor metabolism, promoting angiogenesis and cell adhesion. *AKT1* promote cell growth and inhibit apoptosis. *MMP2* plays a role in inflammation and helps form new blood vessels while *MMP-9* has a function in cell invasion, and metastasis. Similarly, we also found that some tumor suppressor genes and genes involved in apoptosis were differentially expressed. Figure 3 shows the significant increase in the mRNA levels for *PTEN* (Fig. 3A 1.5-fold, *p* = 0.03), *BCL-2* (Fig. 3B, 1.4-fold, *p* = 0.003), and *Caspase-3* (Fig. 3D, 1.3-fold, *p* = 0.04) after transfection with HOXA11-AS. PTEN is a classic tumor suppressor gene while BCL2, BAX and Caspase-3 are regulators of apoptosis. We also examined the expression of ZEB1, ZEB2, Snail1, SnaiL2, E-cadherin, N-cadherin, EZH2 and DNMT1, Stat3, ROCK1, MMP16, LATS1, p21 and KLF2, none of which were significantly different (data not shown). We observed a trend towards increased mRNA levels of BAX but not to a significant level.

## Discussion

Endometriosis is an inflammatory gynecological disorder found among reproductive-age women leading pelvic pain and infertility [32–35]. Endometriosis is a systemic disease with a multitude of effects in many organs [23]. The molecular mechanisms underlying the pathophysiology of endometriosis is not fully characterized. We and others have previously reported that HOXA11-AS regulates endometrial proliferation and inhibits decidualization [22]. Progesterone represses HOXA11-AS, decreasing expression in the secretory phase allowing decidualization [22]. Progesterone resistance, in part mediated by lower levels of progesterone receptor, diminish response to progesterone in endometriosis [36, 37]. We predicted HOXA11-AS would be regulated by progestins and could be diminished by progestin resistance. Here we investigated lncRNA HOXA11-AS expression in women with endometriosis during progestin treatment to determine if HOXA11-AS regulation contributed to the therapeutic effect of progestins.

Our previous study characterized differential expression of lncRNA HOXA11-AS in mouse and human endometrium during menstrual cycle. We reported that HOXA11-AS peaked at the mid-proliferative phase and varied inversely with HOXA11 mRNA expression levels [20, 38]. HOXA11-antisense likely has a role in regulating HOXA11, a gene required for normal uterine development, endometrial receptivity and pregnancy [20, 39, 40]. HOXA11 expression is decreased in endometriosis [41]. Furthermore, we observed that progesterone down-regulated HOXA11-antisense transcription [20]. Similar to a study reported by Wang et al., here we saw no significant differences in HOXA11-AS expression levels in the eutopic endometria between controls without endometriosis and with endometriosis [29]. They also found a significant increase in HOXA11-AS mRNA levels in the ectopic lesions compared to eutopic endometria from women with endometriosis without hormonal treatment. HOXA11-AS likely has an important role in promoting proliferation of ectopic endometrium.

In this study we examined tissue from women with endometriosis who were using hormonal treatments consisting of either COC (combined oral contraceptives) or a progestin alone. Our results revealed a significant decrease in HOXA11-AS levels after hormonal treatment with progestins in ectopic endometrial tissue from women with endometriosis. While both endometrium and ectopic lesions showed decreased HOXA11-AS expression compared to normal proliferative phase levels [20], the endometriosis lesions showed the most profound decrease.

There was no significant difference in HOXA11-AS mRNA expression between endometriosis eutopic and control eutopic tissue in response to progestin treatment. A limitation of this study is the small number of subjects using progestin alone therapy compared to those using a COC; we did not have the power to perform a subgroup analysis to see if these treatments varied. However, we can conclude that reduction of HOXA11-AS expression is a mechanism of progestin action and progestin based therapies.

We next identified genes targeted by HOXA11-AS in endometriosis cells (ENDO), an immortalized cell line by overexpressing HOXA11-AS. Elevations in HOXA11-AS induced here would reflect changes in gene expression that drive disease. LncRNAs can bind directly or indirectly to mRNAs to regulate gene expression. More commonly lncRNAs may acts as competitive endogenous RNAs (ceRNAs) and bind to microRNAs (miRNAs), sequestering them to antagonize the suppression of miRNAs on mRNAs expression. For example, *ACTA2* gene expression is increased by lncRNA H19 which sponges to miR-216a-5p to mediate stromal cell invasion and migration in endometriosis [42] and alters stromal cell growth [43]. Similarly, increases in *CHL1* gene expression reported in endometrial cancer are driven by lncRNA CHL1-AS1, which sponges miR-6076 to promote cell proliferative and migration [44]. *DOLPP1* gene expression is regulated by lncRNA LINC00958 by similarly sponging miR-761 [45]. LncRNA HOTAIR is involved in the development of endometriosis by promoting endometrial stromal cell invasion and migration by increasing *PRRG4* expression through sponging of miR-519b-3p [46]. LcnRNAs play an important regulatory role through their effects on multiple target genes.

Here we identified several targets of HOXA11-AS regulation. We demonstrated higher gene expression of *PTEN*, *BCL2*, *BAX*, and *Caspase 3* in HOXA11-AS transfected ENDO cells. *PTEN* is a tumor suppressor gene while *BCL2*,* BAX* and *Caspase 3* are apoptosis-related genes. A regulatory effect on PTEN expression has been seen with other lncRNAs including the lncRNA C8orf49 and is highly expressed in endometriosis and sponges to microRNA miR-1323 to facilitate p-PTEN-mediated cell proliferation and metastasis of ESCs [47]. Similarly, upregulation of lncRNA Linc-ROR expression in adenomyosis inhibited PTEN function through phosphorylation [48]. Consistent with our findings, increased *BCL-2* gene expression has been reported in ectopic endometrial stromal cells [49]. Similarly, *BCL-2* is also regulated by another non-coding RNA, specifically lncRNA MALAT1 [50].

We also observed an altered expression of several genes that are involved in endometriosis attachment and invasion. Increased expression of *ITGB3*, *AKT1*, *MMP2*, and *MMP9* genes was seen in response to HOXA11-AS overexpression. Several lncRNAs including HOXA11-AS are involved in the pathogenesis of endometriosis and cancer by activating similar genes as demonstrated here [51, 52]. *ITGB3* is upregulated in endometriosis [53]. Similarly, lncRNA EIF3J-AS1 acts as an oncogene and induces *AKT1* expression in esophageal cancer to promote cell invasion and metastasis [54]. Matrix metalloproteinases (MMPs) activity, including MMP2 and MMP9, is elevated in the ectopic tissue of endometriosis [55, 56]. Lnc-216 and *OSTM1-AS1 also* upregulate *MMP2* and *MMP9* in retinal endothelial function and renal cell carcinoma respectively [57, 58]. HOXA11-AS regulates several target genes that are coregulated by multiple lncRNAs, suggesting an intricate network of RNA molecules play a major role in regulating endometriosis.

## Conclusion

HOXA11-AS is a key regulator of endometriosis. Progestins decrease HOXA11-AS expression and therefore its regulation of multiple downstream targets that promote endometriosis. LncRNAs are part of a complex regulatory system in the pathogenesis of endometriosis and are also part of its treatment.

## Electronic supplementary material

Below is the link to the electronic supplementary material.

Supplementary fig. 1: Showing HOXA11-AS-pcDNA3.1(+) plasmid map (7039 bp) containing HOXA11-AS gene (1640 bp)

Supplementary fig. 2: HOXA11-AS expression levels in COC and progestin subgroups. mRNA levels in ectopic lesions were determined by quantitative RT-qPCR from patients with endometriosis. HOXA11-AS mRNA levels were downregulated in endometrium and endometriosis tissues in response to progestin treatment. There were no significant changes in HOXA11-AS mRNA levels between COC and progestin treated groups. Each bar represents the means ± SEM for data from 3 individual experiments, and each experiment was performed in duplicate

Supplementary Material 3

## Data Availability

No datasets were generated or analysed during the current study.
