# Supplementary figures and images for: The role of long non-coding ribonucleic acid HOXA11-AS in endometriosis therapy
Source: Reprod Biol Endocrinol. 2025 Jun 2;23:83. doi: 10.1186/s12958-025-01420-0 (PMC12128536; doi:10.1186/s12958-025-01420-0)

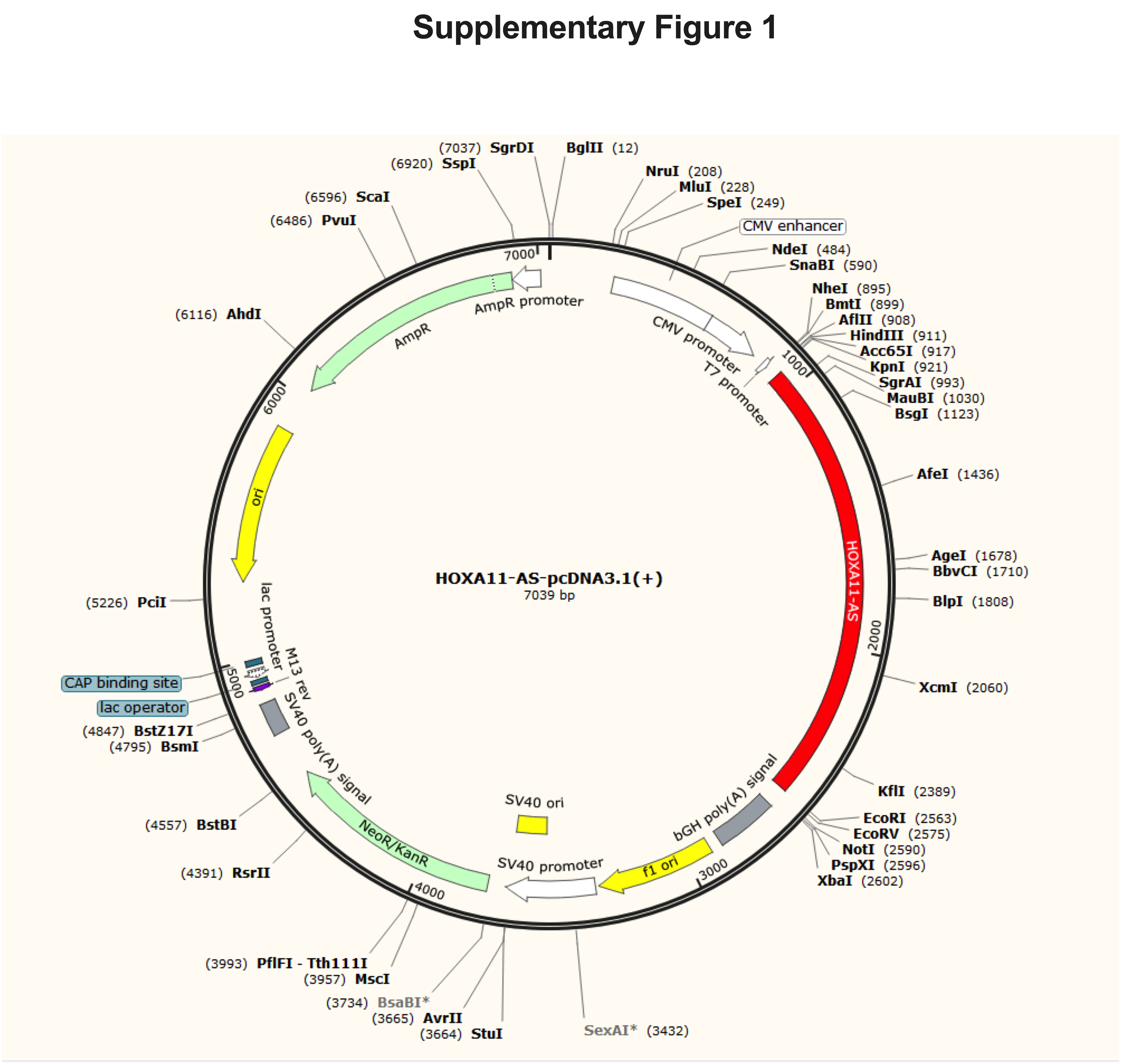

Supplement: Supplementary file 1 — Supplementary fig. 1: Showing HOXA11-AS-pcDNA3.1(+) plasmid map (7039 bp) containing HOXA11-AS gene (1640 bp) [file 12958_2025_1420_MOESM1_ESM.tiff]

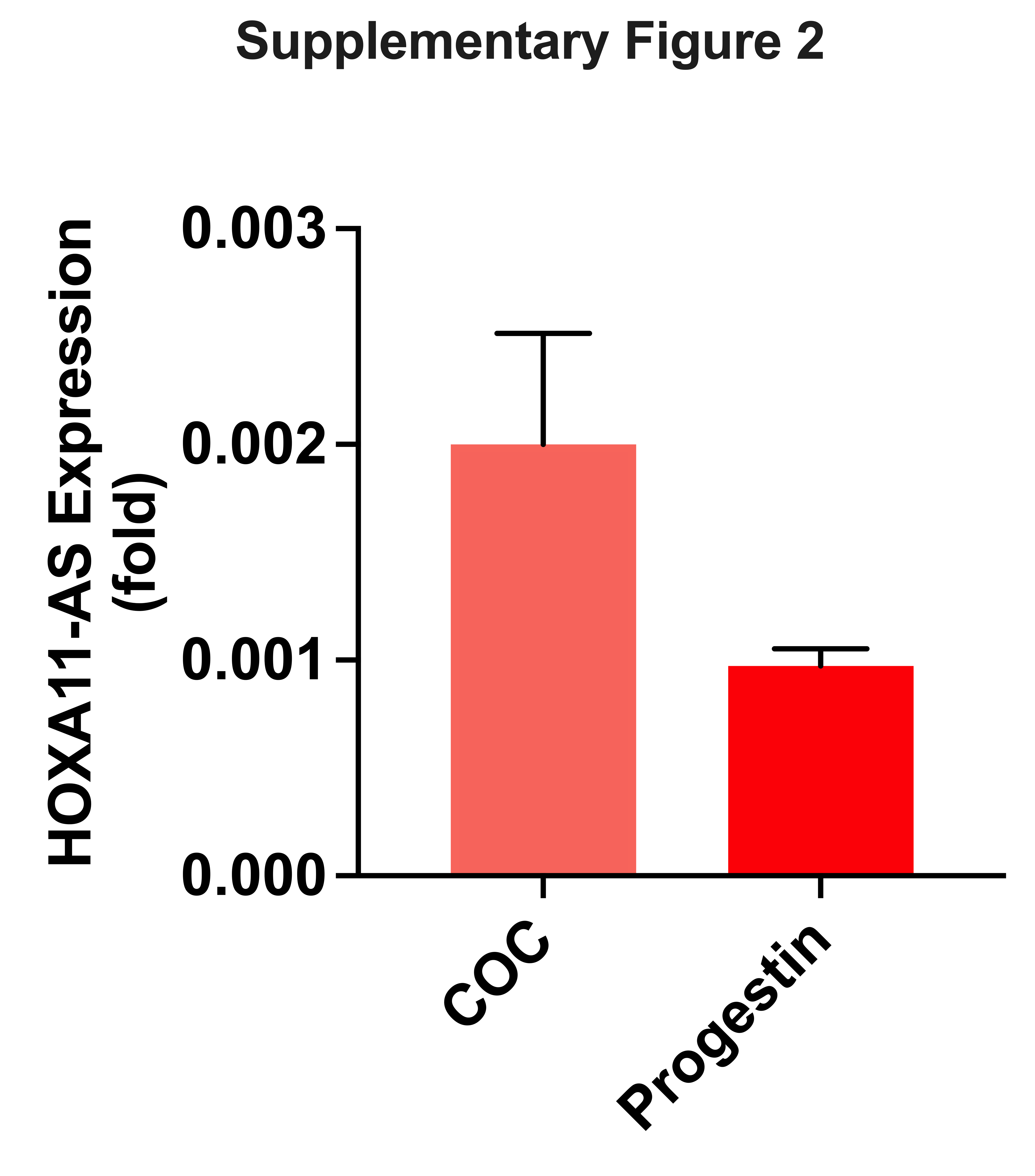

Supplement: Supplementary file 2 — Supplementary fig. 2: HOXA11-AS expression levels in COC and progestin subgroups. mRNA levels in ectopic lesions were determined by quantitative RT-qPCR from patients with endometriosis. HOXA11-AS mRNA levels were downregulated in endometrium and endometriosis tissues in response to progestin treatment. There were no significant changes in HOXA11-AS mRNA levels between COC and progestin treated groups. Each bar represents the means ± SEM for data from 3 individual experiments, and each experiment was performed in duplicate [file 12958_2025_1420_MOESM2_ESM.tiff]
